# A Novel Serum-Free Monolayer Culture for Orderly Hematopoietic Differentiation of Human Pluripotent Cells via Mesodermal Progenitors

**DOI:** 10.1371/journal.pone.0022261

**Published:** 2011-07-27

**Authors:** Akira Niwa, Toshio Heike, Katsutsugu Umeda, Koichi Oshima, Itaru Kato, Hiromi Sakai, Hirofumi Suemori, Tatsutoshi Nakahata, Megumu K. Saito

**Affiliations:** 1 Clinical Application Department, Center for iPS Cell Research and Application, Kyoto University, Kyoto, Japan; 2 Department of Pediatrics, Graduate School of Medicine, Kyoto University, Kyoto, Japan; 3 Laboratory of Embryonic Stem Cell Research, Stem Cell Research Center, Institute for Frontier Medical Sciences, Kyoto University, Kyoto, Japan; 4 Institute of Molecular Medicine, University of Texas Health Science Center, Houston, Texas, United States of America; 5 Waseda Bioscience Research Institute in Helios, Singapore; University of Minnesota, United States of America

## Abstract

Elucidating the *in vitro* differentiation of human embryonic stem (ES) and induced pluripotent stem (iPS) cells is important for understanding both normal and pathological hematopoietic development *in vivo*. For this purpose, a robust and simple hematopoietic differentiation system that can faithfully trace *in vivo* hematopoiesis is necessary. In this study, we established a novel serum-free monolayer culture that can trace the *in vivo* hematopoietic pathway from ES/iPS cells to functional definitive blood cells via mesodermal progenitors. Stepwise tuning of exogenous cytokine cocktails induced the hematopoietic mesodermal progenitors via primitive streak cells. These progenitors were then differentiated into various cell lineages depending on the hematopoietic cytokines present. Moreover, single cell deposition assay revealed that common bipotential hemoangiogenic progenitors were induced in our culture. Our system provides a new, robust, and simple method for investigating the mechanisms of mesodermal and hematopoietic differentiation.

## Introduction

Because of pluripotency and self-renewal, human embryonic stem (ES) cells and induced pluripotent stem (iPS) cells are potential cell sources for regenerative medicine and other clinical applications, such as cell therapies, drug screening, toxicology, and investigation of disease mechanisms [1], [2], [3]. iPS cells are reprogrammed somatic cells with ES cell-like characteristics that are generated by introducing certain combinations of genes, proteins, or small molecules into the original cells [4], [5], [6], [7]. Patient-derived iPS cells have facilitated individualized regenerative medicine without immunological or ethical concerns. Moreover, patient- or disease-specific iPS cells are an important resource for unraveling human hematological disorders. However, for this purpose, a robust and simple hematopoietic differentiation system that can reliably mimic in vivo hematopoiesis is necessary.

Mesodermal and hematopoietic differentiation is a dynamic event associated with changes in both the location and phenotype of cells [8], [9], [10], [11]. Some primitive streak (PS) cells appearing just after gastrulation form the mesoderm, and a subset of mesodermal cells differentiate into hematopoietic cell lineages [9], [12], [13], [14], [15], [16]. Previous studies have accumulated evidence on these embryonic developmental pathways.

The leading methods of blood cell induction from ES/iPS cells employ 2 different systems: monolayer animal-derived stromal cell coculture and 3-dimensional embryoid body (EB) formation. Both methods can produce hematopoietic cells from mesodermal progenitors, and combinations of cytokines can control, to some extent, the specific lineage commitment [1], [2], [17], [18], [19], [20], [21], [22], [23], [24], [25], [26], [27], [28]. In the former method, a previous study showed that OP9 stromal cells, which are derived from the bone marrow of osteopetrotic mice, augment the survival of human ES cell-derived hematopoietic progenitors [29]. However, as the stromal cell condition controls the robustness of the system, it can be relatively unstable. Furthermore, the induction of hematopoietic cells from human pluripotent cells on murine-derived cells is less efficient than that from mice cells.

In EB-based methods, hematopoietic cells emerge from specific areas positive for endothelial markers such as CD31 [30], [31], [32]. Through these methods, previous studies have generated a list of landmark genes for each developmental stage, such as *T* and *KDR* genes for the PS and mesodermal cells, respectively [12], [16], [17], [18], [25], [28], [33], [34], [35], [36], and also have emphasized appropriate developmental conditions consisting of specific microenvironments, signal gradients, and cytokines given in suitable combinations with appropriate timing. For robust and reproducible specification to myelomonocytic lineages of cells, some recent studies have converted to serum-independent culture by using EB formation [37]. However, the difficulty in applying 3-dimensional location information inside EBs prevents substantial increases in hematopoietic specification efficacy. Additionally, the sphere-like structure of the EB complicates tracking and determination of hematopoietic–stromal cell interactions.

To overcome these issues, we established a novel serum-free monolayer hematopoietic cell differentiation system from human ES and iPS cells. Although there are no reports describing the shift of human ES/iPS cells from primitive to definitive erythropoiesis in a monolayer xeno-cell-free condition, our system can trace the in vitro differentiation of human ES/iPS cells into multiple lineages of definitive blood cells, such as functional erythrocytes and neutrophils. Hematopoietic cells arise via an orderly developmental pathway that includes PS cells, mesoderm, and primitive hematopoiesis.

## Materials and Methods

### Maintenance of human ES/iPS cells in serum-free condition

Experiments were carried out with the human ES cell lines KhES-1 and KhES-3 (kindly provided by Norio Nakatsuji) and iPS cell lines 201B7 and 253G4 (kindly provided by Shinya Yamanaka). Stable derivatives of ES cells carrying the transgene for green fluorescent protein (GFP) after CAG promoter were also used [38], [39]. The ES/iPS cells were maintained on a tissue culture dish (#353004; Becton-Dickinson, Franklin Lakes, NJ) coated with growth factor-reduced Matrigel (#354230; Becton-Dickinson) in mTeSR1 serum-free medium (#05850; STEMCELL Technologies, Vancouver, BC, Canada). The medium was replaced everyday. Passage was performed according to the manufacturer's protocol.

### Differentiation of ES/iPS cells

First, undifferentiated ES/iPS cell colonies were prepared at the density of less than 5 colonies per well of a 6-well tissue culture plate (#353046; Becton-Dickinson). When individual colony grew up to approximately 500 µm in diameter, mTeSR1 maintenance medium was replaced by Stemline II serum-free medium (#S0192; Sigma-Aldrich, St. Louis, MO) supplemented with Insulin-Transferrin-Selenium-X Supplement (ITS) (#51500-056; Invitrogen, Carlsbad, CA). This day was defined as day 0 of differentiation. BMP4 (#314-BP-010; R&D Systems, Minneapolis, MN) was added for first 4 days and replaced by VEGF_165_ (#293-VE-050; R&D Systems) and SCF (#255-SC-050; R&D Systems) on day 4. On day 6, the cytokines were again replaced by the haematopoietic cocktail described in the result section. Concentration of each cytokine was as follows: 20 ng/mL BMP4, 40 ng/mL VEGF_165_, 50 ng/mL SCF, 10 ng/mL TPO (#288-TPN-025; R&D Systems), 50 ng/mL IL3 (#203-IL-050; R&D Systems), 50 ng/mL Flt-3 ligand (#308-FK-025; R&D Systems), 50 ng/mL G-CSF (#214-CS-025; R&D Systems), 50 ng/mL complex of IL-6 and soluble IL-6 receptor (FP6) (kindly provided by Kyowa Hakko Kirin Co., Ltd., Tokyo, Japan) and 5 IU/mL EPO (#329871; EMD Biosciences, San Diego, CA). Thereafter, the medium was changed every 5 days.

### Antibodies

The primary murine anti-human monoclonal antibodies used for flow cytometric (FCM) analysis are as follows: PE-conjugated anti-SSEA-4 (#330405; BioLegend, San Diego, CA), Alexa Fluor® 647-conjugated anti-TRA-1-60 (#560122; Becton-Dickinson), biotin-conjugated anti-CD140a (#323503; Biolegend), Alexa Fluor® 647-conjugated anti-KDR (#338909; BioLegend), PE-conjugated anti-CXCR4 (#555974; Becton-Dickinson), PE-conjugated anti-CD117 (#313203; BioLegend), PE-conjugated CD34 (#A07776; Beckman Coulter, Brea, CA), FITC-conjugated CD43 (#560978; Becton-Dickinson), and APC-conjugated CD45 (#IM2473; Beckman Coulter). A streptavidin-PE (#554061; Becton Dickinson) was used as secondary antibody against biotin-conjugated primary antibody. The primary antibodies used to immunostain the colonies and floating blood cells included anti-human Oct3/4 (#611203; Becton-Dickinson), T (#sc-101164; Santa Cruz Biotechnology, Santa Cruz, CA), KDR (#MAB3571; R&D Systems), VE-Cadherin (#AF938; R&D Systems), and rabbit anti-pan-human Hb (#0855129; MP Biomedicals, Solon, OH). FITC-conjugated donkey anti-rat antibodies and Cy3-conjugated goat anti-mouse antibodies (Jackson ImmunoResearch Laboratories, Inc., West Grove, PA) were used as secondary antibodies.

### Cytostaining

Floating cells were centrifuged onto glass slides by using a Shandon Cytospin 4 Cytocentrifuge (Thermo, Pittsburgh, PA) and analysed by microscopy after staining with May–Giemsa or myeloperoxidase. For immunofluorescence staining, cells fixed with 4% paraformaldehyde were first permeabilized with phosphate-buffered saline containing 5% skimmed milk (Becton–Dickinson) and 0.1% Triton X-100 and then incubated with primary antibodies, followed by incubation with FITC or Cy3-conjugated secondary antibodies. Nuclei were counterstained with 4,6-diamidino-2-phenylindole (DAPI) (Sigma-Aldrich).

### Flow cytometric analysis

The adherent cells were treated with Dispase (#354235; Becton-Dickinson) and harvested by gently scraping the culture dish. Aggregated cell structure was chopped by a pair of scissors, processed by GentleMACS (Milteny Biotec, Germany) and then dispersed by 40-µm strainers (#2340; Becton-Dickinson) before staining with antibodies. Dead cells were excluded by DAPI staining. Samples were analysed using a MACSQuant (Milteny Biotec) and FlowJo software (Thermo). Cell sorting was performed using a FACSVantage or FACSAria (Becton–Dickinson).

### RNA extraction and real-time quantitative PCR analysis

RNA samples were prepared using silica gel membrane-based spin-columns (RNeasy Mini-KitTM; Qiagen, Valencia, CA) and subjected to reverse transcription (RT) with a Sensiscript-RT Kit (Qiagen). All procedures were performed according to the manufacturer's instructions. For real-time quantitative PCR, primers and the fluorogenic probes were designed and selected according to Roche Universal Primer library software (Roche Diagnostics) and MGB probe system (Applied Biosystems, Carlsbad, CA). The instrument used was the Applied Biosystems ABIPrism 7900HT sequence detection system, and the software for data collection and analysis was SDS2.3. A GAPDH RNA probe (Hs00266705_g1) was used to normalise the data.

### Clonogenic colony-forming assay

At the indicated days of culture, from days 6 through 25, the adherent cells were treated with dispase and harvested. They were incubated in a new tissue-culture dish (#3003, Becton–Dickinson) for 10 min to eliminate adherent non-haematopoietic cells [40]. Floating cells were collected and dispersed by 40-µm strainers. After dead cells were eliminated by labeling with Dead-Cert Nanoparticles (#DC-001, ImmunoSolv, Edinburgh, UK), live hematopoietic cells were cultured at a concentration of 1×10^3^ (for counting CFU-G) or 10^4^ (for counting CFU-Mix, BFU-E, and CFU-GM) cells/ml in 35-mm petri dishes (#1008; Becton-Dickinson) using 1 ml/dish of MethoCult GF+ semisolid medium (#4435; STEMCELL Technologies) as previously described. Colonies were counted after 14–21 days of incubation, and colony types were determined according to the criteria described previously [41], [42], [43] by in situ observation using an inverted microscope. The abbreviations used for the clonogenic progenitor cells are as follows: CFU-Mix, mixed colony-forming units; BFU-E, erythroid burst-forming units; CFU-GM, granulocyte–macrophage colony-forming units; and CFU-G, granulocyte colony-forming units.

### Single-cell deposition assay

The single-cell deposition assay was performed as described previously [17], [18], [28] [17], [18], [28] [17], [18], [28] [17], [18], [28] [17], [18], [28] [17], [18], [28] [17], [18], [28] [17], [18], [28] [17], [18], [28] [17], [18], [28] [17], [18], [28] [17], [18], [28] [17], [18], [28] [17], [18], [28] [17], [18], [28] [17], [18], [28] [17], [18], [28] [17], [18], [28] [17], [18], [28] [17], [18], [28]. In brief, single sorted cells were deposited in individual wells of 96-well plates with confluent OP9 layers and cultured for 14 days. Wells were scored by morphological observation for hematopoietic colony detection and stained with anti-vascular endothelial cadherin (VE-cadherin) antibodies for endothelial lineage detection.

### Chemotaxis assay

Chemotaxis assay was performed with modified Boyden chamber method using 3.0-µm pore size cell culture inserts (Becton Dickinson). In brief, 5×10^4^ cells harvested from floating cell fraction at day 25 were added to the upper chamber and induced to migrate towards the lower chamber containing 10 nM formyl-Met-Leu-Phe (fMLP; Sigma-Aldrich) for 4 hours at 37°C. After incubation, the cells in the lower chamber were collected and counted using a MACSQuant flow cytometer (Miltenyi Biotec). For quantitative analysis, equivalent amount of 6-µm beads (Becton Dickinson) were added to each FCM sample, and the cell numbers were determined by measuring the ratio of cells to beads.

### Phagocytosis and detection of reactive oxygen species

Phagocytosis and production of reactive oxygen species were detected by chemiluminescent microspheres (luminol-binding carboxyl hydrophilic microspheres; TORAY, Tokyo, Japan) as described previously [44]. In brief, 2×10^4^ floating cells were suspended in 50 µL of the reaction buffer (HBSS containing 20 mM *N*-2-hydroxyethylpiperazine-*N*-2-ethanesulfonic acid (HEPES)) per tube. To activate the system, 5 µl chemiluminescent microspheres were added, and light emission was recorded continuously using a luminometer (TD-20/20; Turner Designs, Sunnyvale, CA). During the measurement, samples were kept at 37°C. To inhibit the reaction, 1.75 µg of cytochalasin B (Sigma-Aldrich) was added to the samples.

### Measurement of oxygen-binding ability

Floating blood cells derived from KhES-1 and 253G4 strains were harvested on day 32 of differentiation (with erythropoietic cytokine cocktail). Oxygen dissociation curves for hemoglobin were measured using a Hemox-Analyzer (TCS Scientific Corporation, New Hope, PA) as previously reported [45], [46].

## Results

### Stepwise generation of functional hematopoietic cells from human ES/iPS cells in the serum-free monolayer culture without animal-derived stromal cells

**Figure 1:**
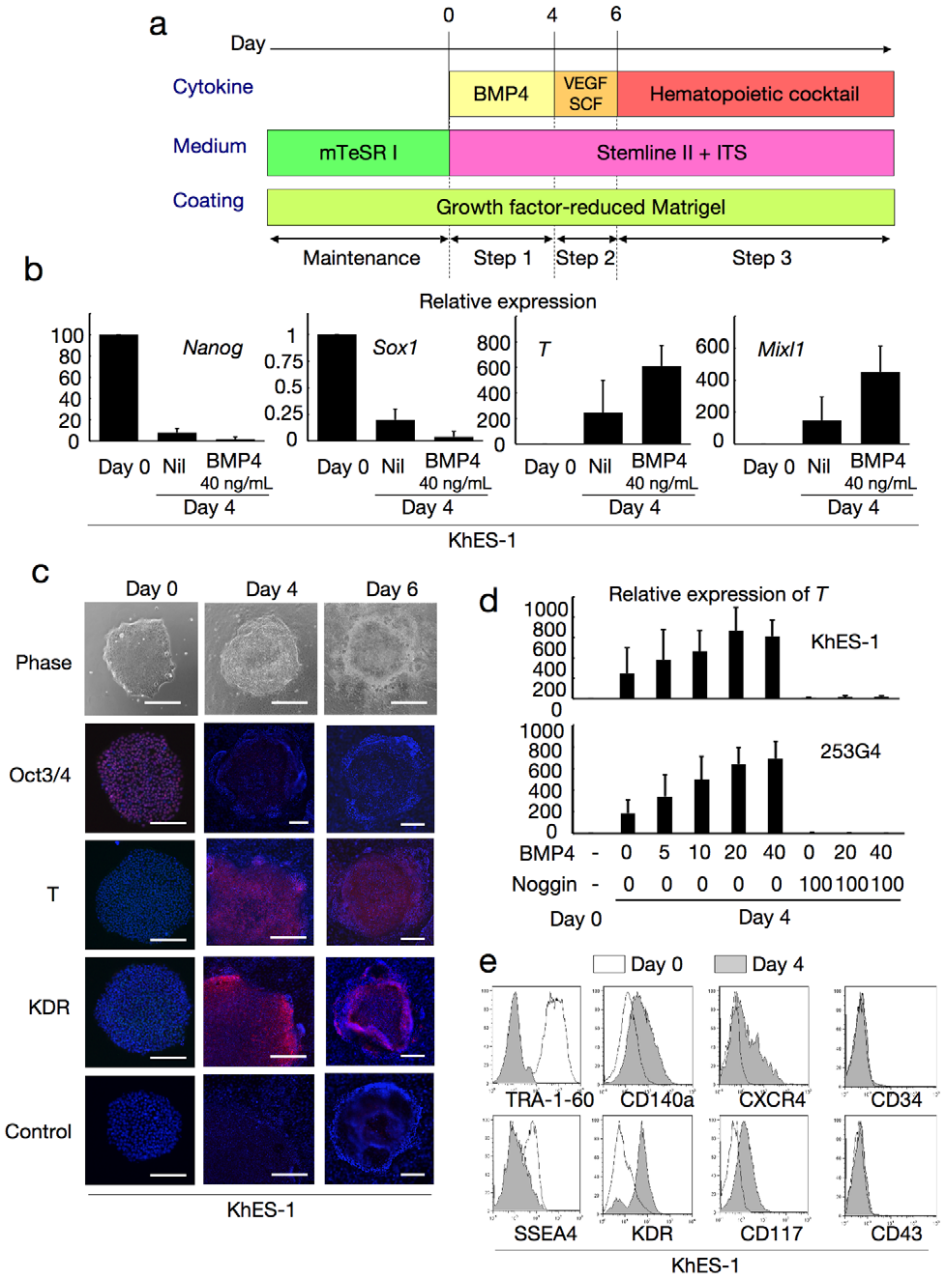
Blood cell induction from pluripotent stem cells starts with commitment into primitive streak. **a.** A schema of stepwise haematopoietic differentiation of human ES/iPS cells. **b.** Gene expression analysis of colonies at the beginning of differentiation (day 0) and the end of step 1 (day 4) with/without 40 ng/mL BMP4. Data from KhES-1 are shown as representative. **c.** Phase contrast microscopies and immunofluorescence staining of colonies during initial differentiation. Data from KhES-1 are shown as representative. **d.** Relative expression of *T* at day 4 of differentiation with different combinations of BMP4 and its inhibitor Noggin. Where shown, bars represent standard deviation of the mean of three independent experiments; Scale bars, 500 µm. Data from KhES-1 and 253G4 strains are shown as representative. **e.** Flow cytometric analysis of differentiating cells on day 4, indicating the down-regulation of immature cell markers and up-regulation of differentiated progenitor markers. Data from KhES-1 are shown as representative.

To assess the differentiation activity of each human ES/iPS cell line with high reproducibility, we used a chemically defined medium in the monolayer differentiation culture and succeeded in inducing various blood cells, including erythrocytes and neutrophils (Figure 1a). To present the developmental process from human ES/iPS cells to blood cells in an orderly manner, we divided the entire process into 3 steps: (1) initial differentiation into PS cells, (2) induction of the hematopoietic mesoderm (Movie S1), and (3) commitment to the hematopoietic lineages (Movie S2).

#### Step 1: Induction of PS-like cells from undifferentiated human ES/iPS cells with BMP4 (days 0–4)

First, we examined the efficacy of initial progression from undifferentiated pluripotent cells (KhES-1) into PS-like cells according to the expression of representative marker genes, such as *T* and *Mixl1* (Figure 1b), and the change in morphology (Figure 1c). Without cytokines, these genes were only slightly upregulated during the first 4 days. However, when 40 ng/mL BMP4 was added to the culture, the expression levels of these genes increased, which is compatible with previous reports on the importance of BMP4 in mesodermal/endodermal differentiation via PS during early embryogenesis (Figure 1b). Further, transcription levels of the undifferentiated marker *Nanog* decreased. During this period, the colonies showed substantial morphological changes at the margins, and cell density decreased and cell contact diminished (Movie S1, Figure 1c). Immunohistochemical staining assays confirmed the upregulation of T and the lateral mesodermal marker, KDR, and downregulation of Oct3/4 (Figure 1c). However, regarding ectodermal commitment, the representative marker gene *Sox1* was hardly detected on day 4 in the presence of BMP4 (Figure 1b).

To assess the role of BMP4 in this step, we analyzed the differentiation efficacy of individual ES (KhES-1 and KhES-3) and iPS (201B7 and 253G4) cell strains with various concentrations of BMP4 in the presence or absence of its inhibitor, Noggin. As shown in Figure 1d, *T* gene expression was upregulated by BMP4 dose-dependently up to 20 ng/mL and was almost completely suppressed by the BMP4 inhibitor, Noggin, in both ES and iPS cells. This suggested that BMP4 was critical at this stage. From these results, we used BMP4 at 20 ng/mL concentration in subsequent experiments.

We also assayed the expression of several cell surface protein markers in this step (Figure 1e). On day 4, undifferentiated cell markers (TRA-1-60 and SSEA4) were downregulated, whereas paraxial and lateral mesoderm cell markers (CD140a and KDR) and markers for mesodermal and hematopoietic progenitors (CXCR4 and CD117) were upregulated. The early-phase hematopoietic-committed cell markers (CD34 and CD43) were still negative at this stage. Similar results were obtained for both ES and iPS cells (data not shown), suggesting that our system initiated paraxial and lateral mesodermal differentiation from pluripotent stem cells during Step 1 [33].

#### Step 2: Generation of KDR^+^CD34^+^CD45^−^ progenitors with VEGF and SCF (days 4–6)

Our previous studies of primate ES cells demonstrated that KDR^+^CD34^+^CD45^−^ mesodermal progenitors derived in a VEGF-containing culture on OP9 stromal cells included hematopoietic progenitors [17], [18]. Therefore, we used this data to induce these progenitors in our culture system. Considering the partial expression of KDR and CD117 during the first step, we replaced BMP4 with 40 ng/mL VEGF_165_ (ligand for KDR) and 50 ng/mL SCF (ligand for CD117) on day 4 to accelerate selective differentiation to the lateral mesoderm with hematopoietic activities (Figure 1a).

**Figure 2:**
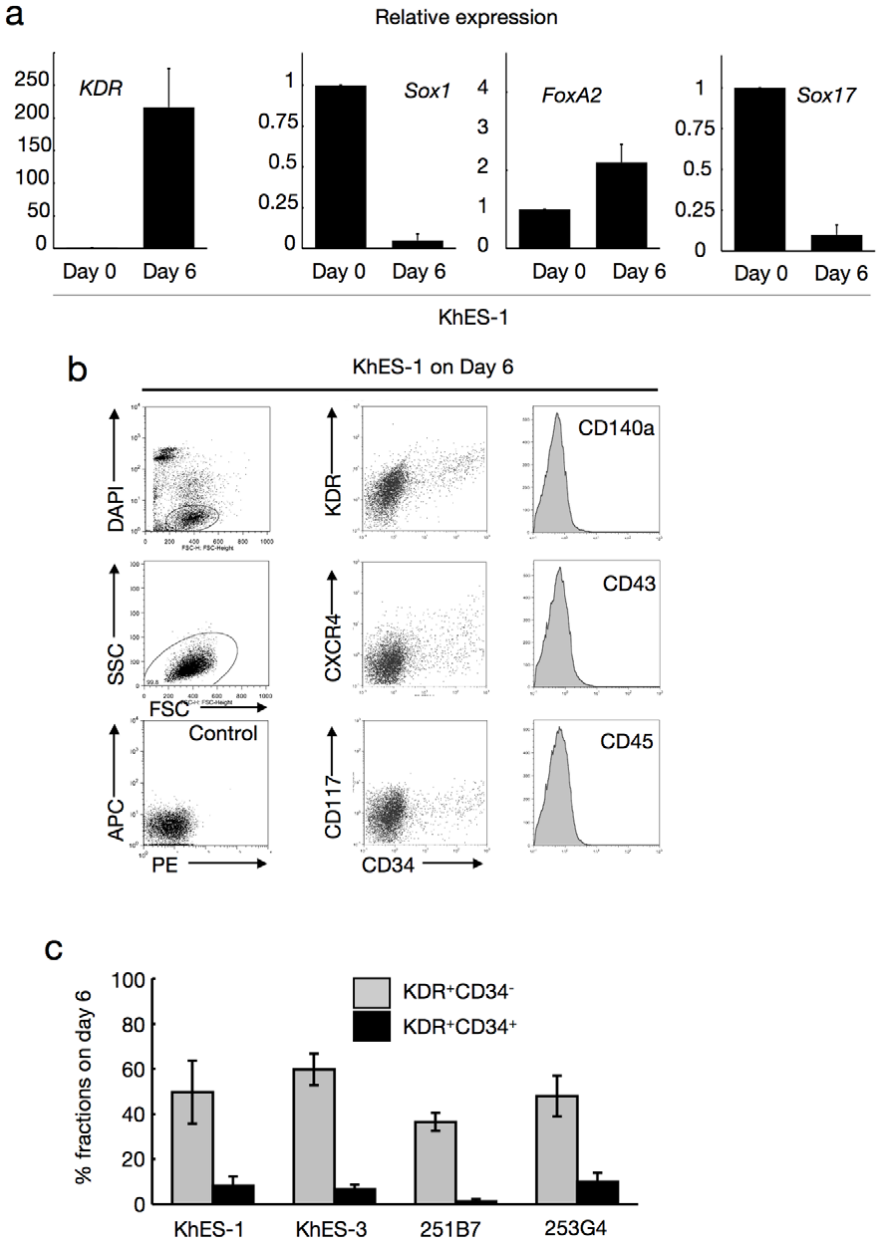
Characterization of cells during initial differentiation with lineage-specific marker expression. **a.** Expression analysis of lineage-specific marker genes at the beginning of differentiation (day 0) and the end of step 2 (day 6). Bars represent standard deviation of the mean of three independent experiments. Data from KhES-1 are shown as representative. **b.** The development of progenitors on day 6 positive for lateral mesoderm markers but negative for paraxial mesoderm and haematopoietic cell markers. Leftmost column shows the gating strategy for eliminating dead cells and debris. Data from KhES-1 are shown as representative. c. Efficacy of inducing KDR^+^CD34^+ or −^ mesodermal progenitors from each two lines of human ES cells and iPS cells. Bars represent standard deviation of the mean of three independent experiments.

During the next 2 days, the colonies exhibited 2 distinct regions: a plateau-like central area with stratified components and a surrounding area with monolayer cells (Movie S1, Figure 1c). On day 6, the mRNA expression pattern indicated the dominance of mesodermal cells rather than endodermal or ectodermal lineages (Figure 2a), and flow cytometric (FCM) analysis demonstrated the emergence of new cell fractions that were positive for KDR, CD117, CXCR4, and CD34 but negative for CD140a, CD43, and CD45 (Figure 2b). Our system robustly supports mesodermal induction from both ES and iPS cells, despite differences in efficacy among cell strains (Figure 2c).

Further, immunohistochemical staining for KDR indicated an uneven distribution of KDR^+^ cells at the marginal zone of the plateau area (Figure 1c), suggesting that differentiation polarity within the colonies resulted in site-specific emergence of putative hematopoietic mesodermal progenitors.

#### Step 3: Production of functional blood cells dependent on cytokine cocktails (day 6 onward)

On day 6, we changed the culture medium to another chemically defined medium containing hematopoietic cytokines (Figure 1a). To achieve lineage-directed differentiation, we used 2 combinations of cytokines: a myeloid-induction cocktail containing SCF, TPO, IL3, FLT-3 ligand, and G-CSF; and an erythropoietic-differentiation cocktail containing SCF, TPO, IL3, FP6, and EPO.

**Figure 3:**
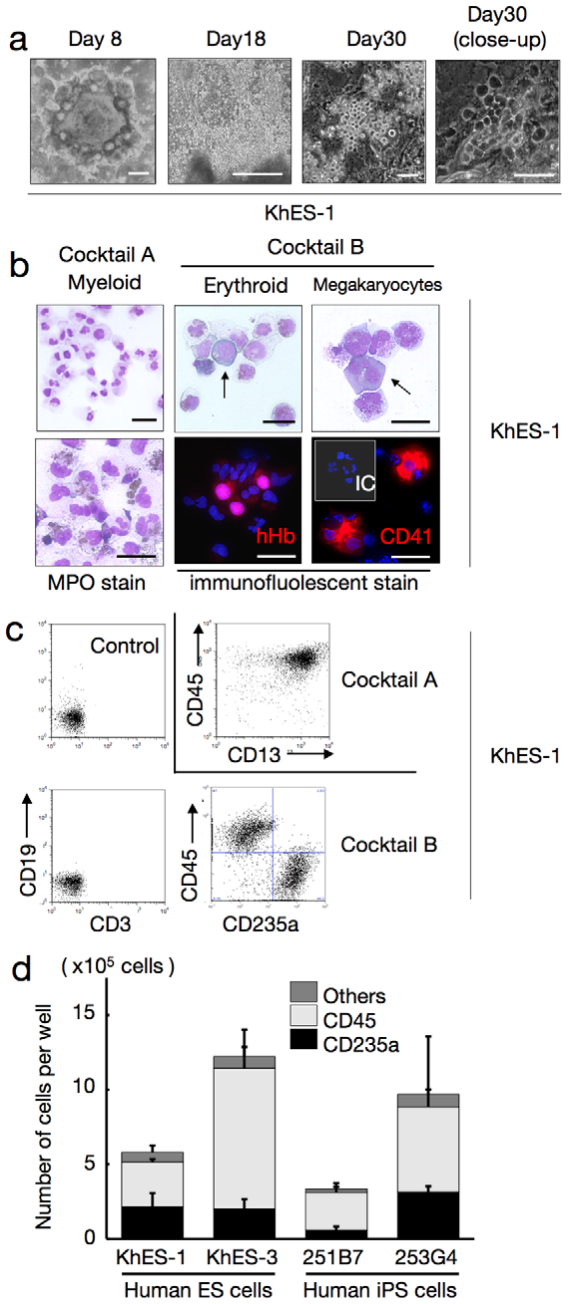
Human ES/iPS cell-derived haematopoiesis in a monolayer culture free from animal serum or stromal cells. **a.** Sequential phase contrast pictures showing haematopoietic development. Scale bars, 500 µm (left two panels) and 100 µm (right two panels). Data from KhES-1 are shown as representative. **b.** Floating cells harvested on day 30 showing various lineages of haematopoietic cells; MPO-positive myeloid lineage cells (leftmost panels), pan-human Hb-positive erythroid lineage cells (centre panels), and CD41-positive megakaryocytes (rightmost panels). Scale bars, 100 µm. Data from KhES-1 are shown as representative. **c.** Expression of lineage-specific antigens on floating cells harvested on day 30; Myeloid lineages (CD13 and CD45), erythroid lineages (CD235a), T cells (CD3), and B cells (CD19). Data from KhES-1 are shown as representative. **d.** Numbers and fraction of blood cells induced from each two lines of human ES cells and iPS cells. Bars represent standard deviation of the mean of three independent experiments.

Regardless of the cocktails, the colonies first exhibited a rosary-like appearance, with small sac-like structures aligned along the margins of the plateau areas, and grew for several days (Figure 3a, left panel). Hematopoietic cell clusters emerged from the edge of these structures on days 10–12, followed by the appearance of floating blood cells a few days later, which increased thereafter; hematopoietic clusters grew in size and number, and some exhibited areas with a cobblestone-like appearance (Figure 3a, right 3 panels; Movie S2). When fresh medium with the cytokines was supplied every 5 days, blood cell production was observed in both ES and iPS cell experiments until day 50 of differentiation, whereas few hematopoietic cells appeared without the cytokines (data not shown).

As expected, the myeloid-induction cocktail induced myelomonocytic lineages predominantly positive for CD45. Blood cells harvested on day 30 exhibited morphology compatible with myelomonocytic precursors and mature neutrophils, and displayed positive myeloperoxidase staining (Figure 3b). On the other hand, the erythropoietic-differentiation cocktail yielded cell lineages that included hemoglobin-positive (Hb^+^) erythroid cells and CD41^+^ megakaryocytes (Figure 3b). In the KhES-1 strain (3.5 [standard deviation (SD) = 1.5] undifferentiated colonies 250 µmm in diameter were initially plated in individual wells of 6-well plates at the start of differentiation), counting and FCM analysis of harvested blood cells on day 30 revealed the existence of 7.7×10^5^ (SD = 2.3×10^5^) different cell lineages per well, including 36.0% (SD = 6.4%) CD235a^+^ erythroid and 53.2% (SD = 9.4%) CD45^+^ myelomonocytic lineages, but no lymphoid lineage cells (Figure 3c). Although the differentiation efficacy and lineage distribution depend not only on the cytokines but also on the cell strains, the data indicates that human ES and iPS cells develop into various lineages of hematopoietic cells, robustly and orderly, in our novel monolayer culture system without xeno-derived serum or stromal cells (Figure 3d).

### ES/iPS cell-derived hematopoietic cells have similar potential to in vivo-derived blood cells in function

**Figure 4:**
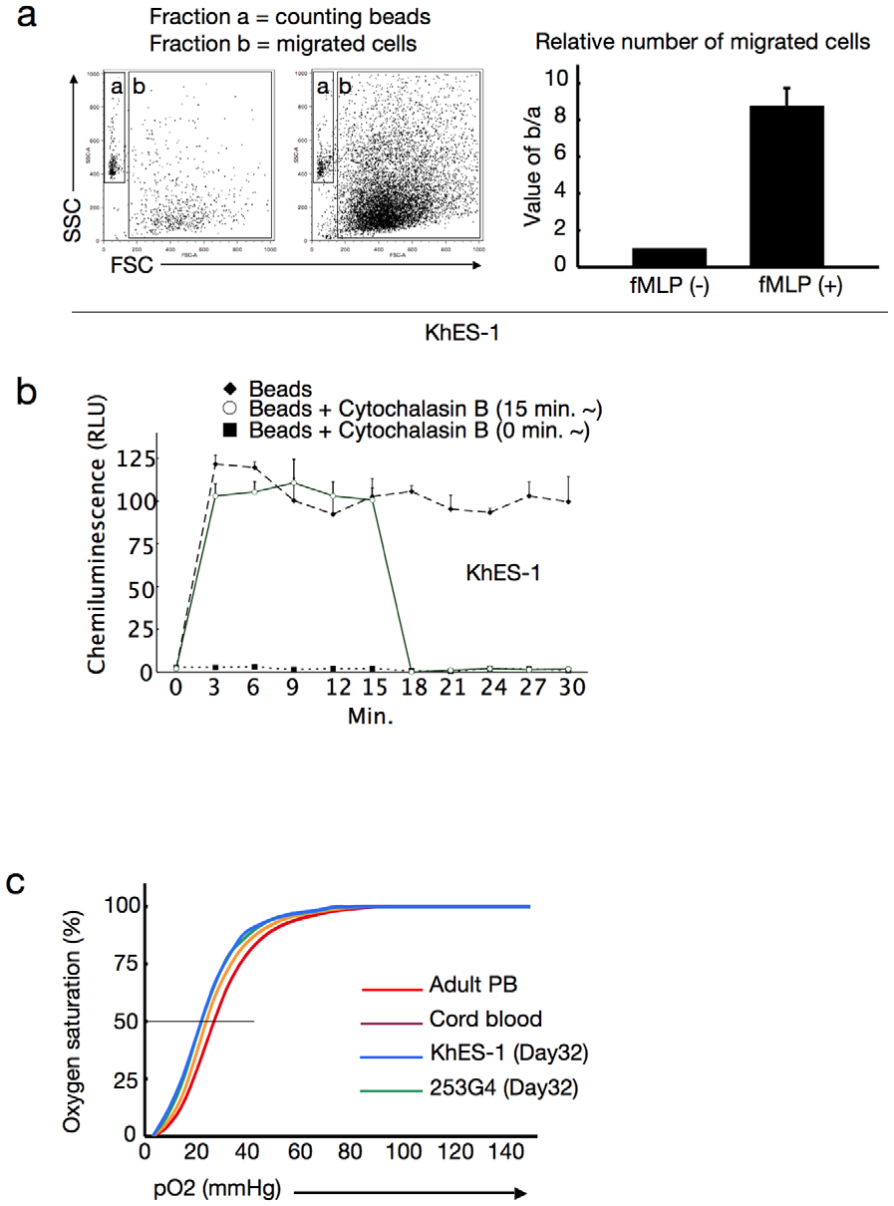
Functional blood cells derived from human ES/iPS cells. **a.** Number of migrated cells that permeated through the transwell membrane with or without fMLP. Values were normalised to the number of counting beads, and the control values were arbitrarily set to the condition without fMLP. Data from KhES-1 are shown as representative. **b.** Assay for phagocytosis-induced respiratory burst activity using chemiluminescent microspheres (luminol-binding microspheres). Abbreviation: RLU, relative light units. Data from KhES-1 are shown as representative. **c.** Oxygen dissociation curves of erythroid cells derived from human ES/iPS cells (harvested on day32 of differentiation), human cord blood, and adult peripheral blood. Where shown, bars represent standard deviation of the mean of three independent experiments.

Considering the use of ES/iPS cell-derived hematopoiesis for various clinical and research applications, it is important to confirm the function of the generated blood cells. Neutrophils derived with the myeloid-induction cocktail exhibited migration activity in response to the chemoattractant fMLP (Figure 4a) and phagosome-dependent reactive oxygen production, which was inhibited by the phagosome destruction agent, cytochalasin B (Figure 4b). On the other hand, erythroid lineage cells derived with the erythropoietic-differentiation cocktail (harvested on day 32 of differentiation) exhibited an oxygen dissociation curve that was similar, despite being slightly left-shifted, to those obtained with adult and cord blood cells (Figure 4c). These data indicate that our culture facilitates robust and orderly development of human ES and iPS cells into functional hematopoietic cells with similar potential to in vivo-derived blood cells.

### Clonogenic hematopoietic development from human ES/iPS cell-derived progenitors

The human hematopoietic system is a hierarchy of various component cells from stem or progenitor cells to terminally differentiated cells. For example, CD34^+^ cells in umbilical cord blood or bone marrow contain putative hematopoietic stem cells and are used as a source of stem cell transplantation. The identification and proliferation of such cells in vitro have been of great interest in medical science research.

**Figure 5:**
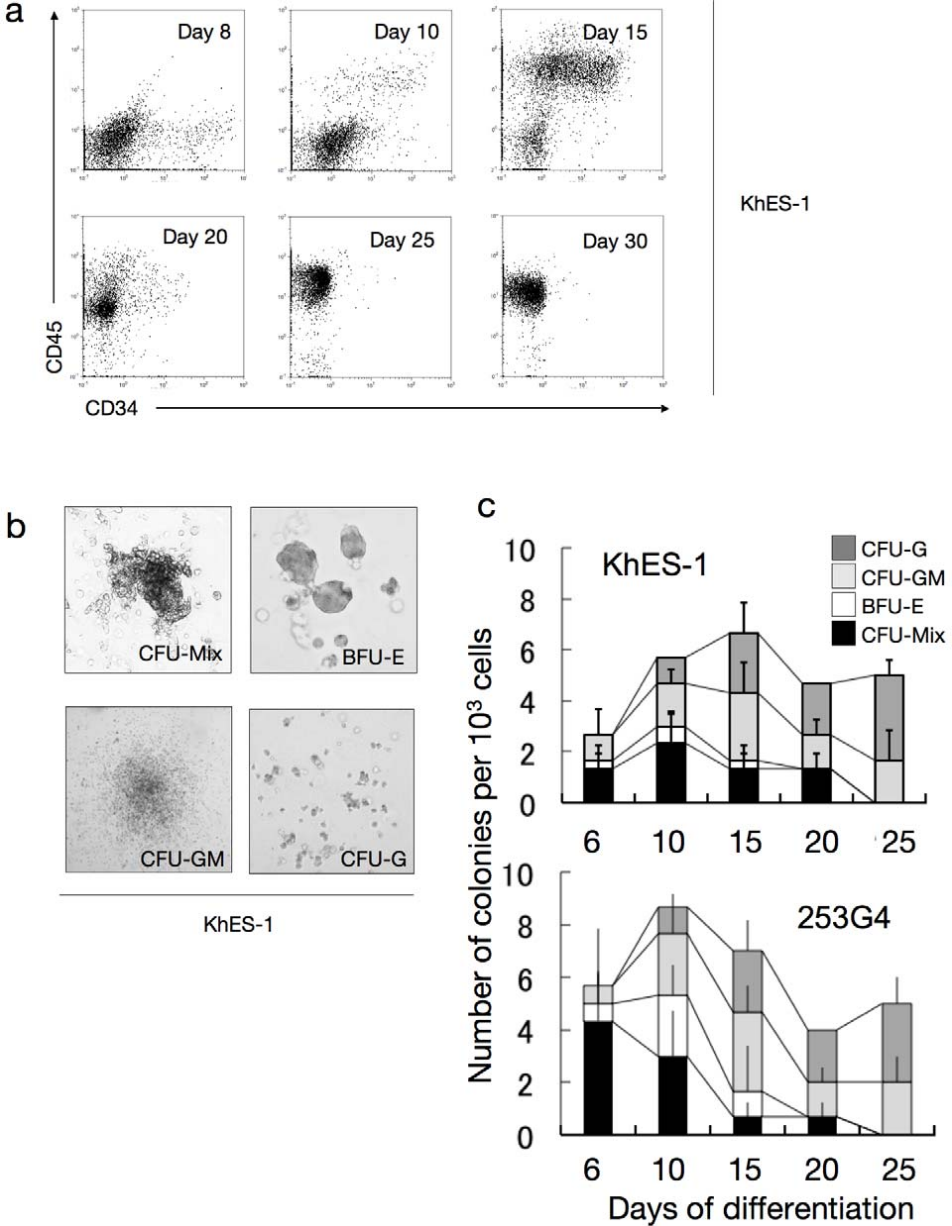
Hematopoietic stem/progenitor cells in culture. **a.** Sequential FCM analysis of cells harvested on indicated days showing the existence of CD34^+^CD45^+^ haematopoietic progenitor cells in culture. Data from KhES-1 are shown as representative. **b.** Various colony types on MTC-containing medium clonally emerged from single haematopoietic progenitor cells. Data from KhES-1 are shown as representative. **c.** Numbers of each colony type derived from different days of culture. Bars represent standard deviation of the mean of three independent experiments. Data from KhES-1 and 253G4 strains are shown as representative.

To assess the potential of our system for supporting generated immature stem or progenitor cells, we evaluated the colony-forming ability of the cultivated hematopoietic progenitors in the system. Accordingly, the cells were cultured with SCF, TPO, IL3, FLT-3 ligand, and FP6. In these conditions, CD34^+^CD45^+^ hematopoietic cells existed up to day 25, indicating that the immature hematopoietic cells can be maintained in our serum-free culture (Figure 5a).

We harvested adherent blood cells from the previously described culture and transferred them into a methylcellulose-containing medium to perform colony-forming assays with SCF, TPO, IL3, G-CSF, and EPO. As shown in Figure 5b and c, CFU-Mix, BFU-E, CFU-GM, and CFU-G colonies developed from plated cells. The total number of colonies increased dramatically from day 6 to day 10, then gradually increased until day 15 and decreased thereafter. CFU-Mix and BFU-E colonies were mainly observed until day 15 and were thereafter replaced by CFU-GM and CFU-G colonies. Similar tendencies were observed in both ES and iPS cells. These results suggest that our culture system can incubate multipotent hematopoietic stem or progenitor cells over a period of time.

### Identification of KDR^+^CD34^+^CD45^−^ bipotential hemoangiogenic progenitors derived in serum-free conditions

**Figure 6:**
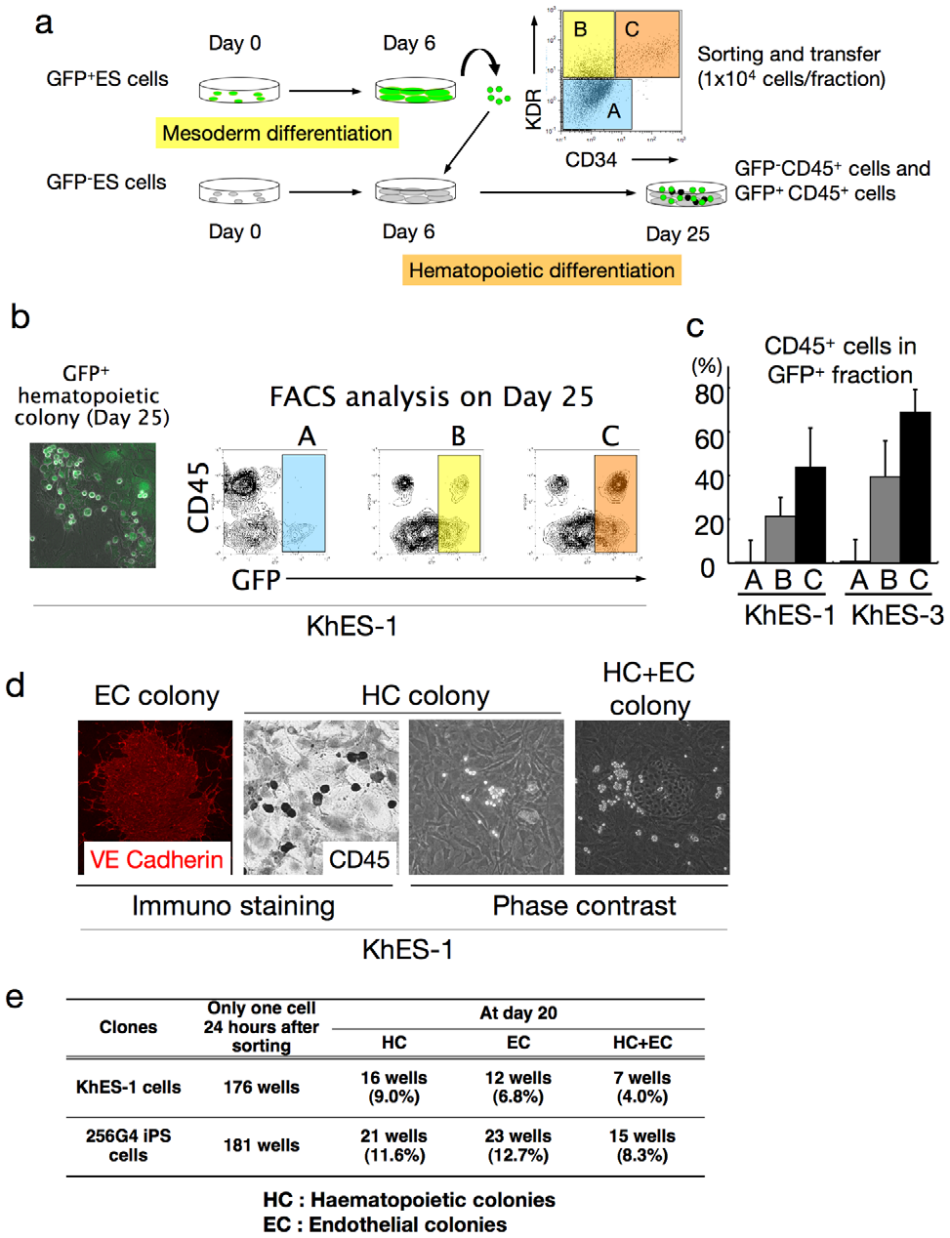
Haematopoietic differentiation from KDR^+^CD34^+^ mesodermal progenitors. **a.** Schema of the protocol for measuring haematopoietic activities of depicted fractions on day 6. **b.** Each sorted fraction-derived haematopoiesis on day 25 detected by fluorescent microscopy and FCM analysis. Data from KhES-1 are shown as representative. **c.** Ratio of CD45^+^ cells in GFP^+^ fraction on day 25 showing the strongest haematopoietic activity of fraction C followed by fraction B. **d.** Single KDR^+^CD34^+^CD45^−^ cell-derived haematopoietic colonies (HC), VE-cadherin^+^ endothelial colonies (EC), and HC+EC colonies generated on OP9 cell layers. Data from KhES-1 are shown as representative. **e.** Number of wells that showed HC, EC, and EC+HC development.

During embryogenesis, hematopoietic development is closely associated with endothelial lineage commitment [47], [48], and previous studies have demonstrated that ES cells can differentiate into the common multipotent progenitors that differentiate into both blood and endothelial cells at the single cell level on OP9 stroma [17], [28], [49]. Although the experiments described thus far demonstrated that the serum-free, xeno-cell-free culture condition supported human ES/iPS cell-derived hematopoiesis in an orderly manner, as observed during embryogenesis, it was unclear which day 6 fraction(s) developed into blood cells. To clarify this point, human ES cells stably expressing green fluorescent protein (GFP) were cultured, then 1×10^4^ cells of GFP^+^KDR^−^CD34^−^CD45^−^ (Fraction A), GFP^+^KDR^+^CD34^−^CD45^−^ (Fraction B), and GFP^+^KDR^+^CD34^+^CD45^−^ (Fraction C) fractions were transferred on day 6 into a synchronous differentiation culture of unlabeled ES cells (Figure 6a). Nineteen days later (day 25 of differentiation), GFP^+^ small round cell-containing colonies were observed predominantly in Fractions B and C, and FCM analysis of the entire culture confirmed the emergence of GFP^+^CD45^+^ cells mainly from Fraction C (Figure 6b). On the other hand, few blood cells positive for GFP were generated from Fraction A. These results were obtained with 2 independent strains of human ES cells (KhES1-EGFPneo on KhES-1 and KhES3-EGFPneo on KhES-3) (Figure 6c) and indicated that hematopoietic progenitors were present in the KDR^+^ fraction, particularly in the KDR^+^CD34^+^ fraction, on day 6 of differentiation.

Finally, we performed a single-cell deposition assay by transferring single sorted human ES/iPS cell-derived GFP^+^KDR^+^CD34^+^CD45^−^ cells, which were negative for VE-cadherin, on day 6 into individual wells of 96-well plates coated with an OP9 cell layer. As shown in Figure 6d and e, the proportion of hematopoietic cell (HC) development, VE-cadherin^+^ endothelial cell (EC) development, and HC plus EC development on day 20 were 9.0%, 6.8%, and 4.0%, respectively, for KhES-1 and 11.6%, 12.7%, and 8.3%, respectively, for 253G4 iPS cells. These results demonstrate that the common mesodermal progenitors that can differentiate into both blood and endothelial cells at the single-cell level are induced in our culture condition.

## Discussion

In this study, we demonstrated the orderly mesodermal and hematopoietic differentiation of human ES and iPS cells in a novel serum-free monolayer culture condition. Simple manipulation of cytokine combinations facilitated robust, reproducible, and highly directed stepwise commitment to specific lineages of functional blood cells.

There are several reports on hematopoietic differentiation of human ES/iPS cells, such as murine-derived OP9 stromal cell coculture and feeder/serum-free EB formation systems [20], [22], [23], [24], [30], [31], [32], [50]. However, two-dimensional cultures containing xeno-serum/cells often cause dependency on their lots, while complicated three-dimensional structures inside EBs make it difficult to assess and control conditions for inducing specific progenitors. Actually, few in vitro systems have been able to reliably reproduce hematopoietic development from mesodermal progenitors or model the in vivo coexistence of developing hematopoietic cells and their autologous microenvironments in serum-free conditions. Our less labor-intensive and clearly defined monolayer culture facilitates observation of the stepwise development of pluripotent cells to blood cells via common hemoangiogenic progenitors and the behavior of hematopoietic cells on autologous stromal cells. Consequently, assays for elucidating differences in lineage specification of various ES/iPS cell strains, including hematopoietic potential, can be performed with high reproducibility. This is particularly important because individual pluripotent cell strains vary in differentiation potentials [51], [52], [53]. This study demonstrated quantitative differences in hematopoietic differentiation efficacy and lineage commitment among 4 ES/iPS cell strains.

Because human ES/iPS cells are feasible cell sources for various clinical applications, scientific and medical communities have shown continuing interest in hematopoietic stem cell induction from ES/iPS cells. Previous trials have indicated that murine ES cell-derived hematopoietic cells overexpressing HoxB4 [54] can replenish the bone marrow of lethally irradiated recipient mice. However, it remains a challenge to develop bona fide human hematopoietic stem cells with bone marrow reconstitution activity at the single-cell level. In our study, we observed many cobblestone area-forming cells, which reportedly indicate the existence of very immature hematopoietic progenitors. Moreover, FCM analyses and colony-forming assays suggested that ES and iPS human cell-derived hematopoiesis in our method occurs through clonogenic hematopoietic stem/progenitor cells. We are in the process of determining in vivo repopulating ability of cells harvested from our culture by using serial transplantation into immunodeficient mice to assess the possibility of inducing feasible cell sources for various clinical applications, such as cell therapies and disease investigation.

Finally, time-lapse imaging strongly indicated crosstalk between hematopoietic cells and the autologous microenvironment composed of non-hematopoietic cells. Emerged blood cells move about actively and generate colonies in surrounding cell layers, suggesting the importance of a direct interaction between blood cells and microenvironmental cells for the maintenance, proliferation, and differentiation of stem or progenitor cells (Movie S3). In fact, a model of hematopoietic disorders triggered by mutation in the bone marrow microenvironment has been recently reported [55]. However, further investigation is necessary to identify the mechanisms responsible for such phenomena. Our culture may aid these investigations as it facilitates simple and sequential harvest of hematopoietic cells with minimal contamination by autologous adherent cell layers.

In conclusion, this study presents novel methods for analyzing the mechanisms of normal hematopoiesis in a robust, reproducible, and stepwise manner. Furthermore, employing gene-manipulated ES cells or disease-specific iPS cells will supply in vitro models of disease pathology, thereby providing further insights into hematological defects in conditions such as aplastic anemia and myelodysplastic syndromes.

## Supporting Information

Movie S1 **Time-lapse microscopic movie showing the morphological change in a single colony from day 0 to day 6 (initial differentiation).** In this period, a colony begins forming a rosary-like morphology as it differentiates. The pictures were automatically taken every 8 minute by Biostation IM (Nikon Instruments, Tokyo, Japan).
(MOV)

Movie S2 **Time-lapse microscopic movie showing the morphological change in a single colony from day 6 to day 25 (hematopoietic differentiation).** After adding hematopoietic cytokines on day 6, hematopoietic cells first emerge from the areas near the edge of stratified zone. The pictures were automatically taken every 8 minute by Biostation IM (Nikon Instruments, Tokyo, Japan).
(MOV)

Movie S3 **Close-up time-lapse microscopic movie showing hematopoietic cells moving about and generating colonies in surrounding cell layers.** The pictures were automatically taken every 8 minute by Biostation IM (Nikon Instruments, Tokyo, Japan).
(MOV)
